# Esthetic Assessment following Ridge Augmentation, Late Implant Placement and Immediate Esthetic Reconstruction of the Atrophic Anterior Maxilla

**DOI:** 10.3390/ijerph19063689

**Published:** 2022-03-20

**Authors:** Sarit Naishlos, Vadim Reiser, Helena Zelikman, Joseph Nissan, Daya Masri, Hiba Nassra, Gavriel Chaushu, Sigalit Blumer, Liat Chaushu

**Affiliations:** 1 Department of Pedodontics, School of Dental Medicine, Tel Aviv University, Tel Aviv 6997801, Israel; river554@gmail.com (S.N.); sigalit@tauex.tau.ac.il (S.B.); 2 Department of Oral and Maxillofacial Surgery, School of Dental Medicine, Tel Aviv University, Tel Aviv 6997801, Israel; vadik.raiser@gmail.com (V.R.); dr.dayamasri@gmail.com (D.M.); gabi.chaushu@gmail.com (G.C.); 3 Department of Oral Rehabilitation, The Maurice and Gabriela Goldschleger School of Dental Medicine, Tel Aviv University, Tel Aviv 6997801, Israel; helenapl@gmail.com (H.Z.); nissandr@gmail.com (J.N.); heba.n93@gmail.com (H.N.); 4 Department of Periodontology and Implant Dentistry, School of Dental Medicine, Tel Aviv University, Tel Aviv 6997801, Israel

**Keywords:** PES/WES, immediate loading, allograft, bone-block

## Abstract

**Purpose**: Evaluate the esthetic outcome of ridge augmentation using cancellous bone-block allografts, late implant placement, and immediate loading in the atrophic anterior maxilla, by PES (pink esthetic score) and WES (white esthetic score) indexes. **Materials and Methods**: Retrospective cohort study. Inclusion criteria were bone loss of at least 3 mm horizontally and 3 mm vertically according to preliminary CBCT; ridge augmentation using cancellous bone-block allografts; six months later the implant insertion and immediately loaded. PES-WES index was used for esthetic assessment of soft tissues surrounding the final implant-supported prosthesis (ISP). **Results**: All twenty-five successive individuals were included. The mean follow-up was 12.1 ± 56 months (range, 42–90 months). The mean PES index and WES index were 7 ± 1.74 (range: 5–10) and 8.4 ± 2.12 (range: 5–10), respectively. The mean total combination of PES index and WES index (PES/WES) was 15.3 ± 2.85 (range: 12–20). All ISPs had an overall score >12 (the defined threshold of clinical acceptability). **Conclusions**: Ridge augmentation in the atrophic anterior maxilla using cancellous bone-block allografts and immediate loading allows a stable esthetic result of the soft and hard tissues over the years (follow-up of 42–90 months).

## 1. Introduction

Esthetic assessment, after rehabilitation of the anterior maxilla with implant-supported prosthesis (ISP), involves the surrounding soft and hard tissues and the prosthesis itself [1]. The optimal position of the implant is 2–4 mm palatal to the buccal bone profile [2]. Objective criteria, PES and WES (Pink Esthetic Score and White Esthetic Score), were developed to assess the esthetics of ISP in the anterior maxilla [3,4,5].

After tooth loss, gradual resorption of the alveolar bone occurs [6,7] in both horizontal and vertical dimensions and might affect the esthetic outcome [7]. Moreover, it may be difficult to install a future implant due to insufficient bone volume [8]. Sometimes, it is necessary to perform bone grafting prior to implant placement [9,10]. A bone-block graft in the anterior maxilla may be recommended [10].

Autogenous bone grafting is not considered the gold standard for bone augmentation anymore [10,11,12,13]. Disadvantages include patient morbidity, financial costs, prolongation of surgery, and patient reluctance for bone harvesting [10]. Furthermore, extensive bone resorption has been reported [11]. A bone graft from an allogeneic source is an alternative [12,13]. Its advantages include reduced patient morbidity and avoidance of an additional surgical procedure (donor site), with shorter surgery duration. The shape of the allogeneic block can be adapted to suit the desired height and width [11]. Bone resorption of allogenic blocks at the time of implant insertion is significantly lower (10–14%) compared to autogenous blocks [11]. The availability of increased bone volume is important, allowing a proper stress distribution for the implant-supported restorations [14].

Treatment time with the standard ISP protocol is long [15,16]. Immediate implant reconstruction is defined as a situation in which at the end of implant placement, a temporary reconstruction is installed [17]. Advantages include immediate function and esthetics, shortening treatment duration, avoiding a second surgical stage, and good adaptation of the gingiva, which may result in less recessions [17].

One of the most important criteria influencing the esthetic result of an ISP is a sufficient thickness of the buccal bone [18,19]. Bone augmentation of the anterior atrophic maxilla using cancellous block-allograft and late loading resulted in predictable esthetic [20]. Immediate function of the ISP could have esthetical implications. Lower papilla index scores were reported for immediate loading ISPs [21].

Long-term evaluation data are still scarce in literature when ridge augmentation using cancellous bone-block allografts are employed. The present study assessed esthetic outcome of ISP located in the anterior atrophic maxilla following extensive bone augmentation using cancellous block allograft and immediate esthetic reconstruction.

## 2. Methods and Materials

The study was approved by the Ethics Committee of Tel Aviv University no. 2137-1. The patients were treated by one oral rehabilitation specialist and one oral and maxillofacial surgeon.

**Inclusion criteria for the study:**

1. Missing teeth in the anterior maxilla characterized by extensive bone loss of at least 3 mm horizontally and at least 3 mm vertically according to primary cone beam computerized tomography (CBCT).
2. Bone augmentations using cancellous allogeneic bone-blocks.
3. Six months later, implant insertion followed immediately by a temporary restoration placement.
4. Age > 18 years.
5. No active periodontal disease (after initial preparation).
6. Existing posterior occlusal support and antagonist natural dentition.
7. Implant insertion at bone level.
8. Similar permanent ISP metal-free porcelain crown type performed in the same laboratory.
9. Periodic follow-up once a year.

**Exclusion criteria included:**

1. Unbalanced systemic diseases (e.g., diabetes).
2. History of radiation to the head and neck area.
3. Poor oral hygiene. (plaque index > 20%).
4. Diseases of the mucous membranes (e.g., lichen planus).
5. Parafunction (e.g., bruxism).
6. Use of medications (e.g., bisphosphonates, calcium channels blockers as amlodipine, hydantoin, cyclosporine).
7. Untreated and uncontrolled periodontal disease.
8. Heavy smoking >10 cigarettes a day.

**Surgical procedure**

**Figure 1:**
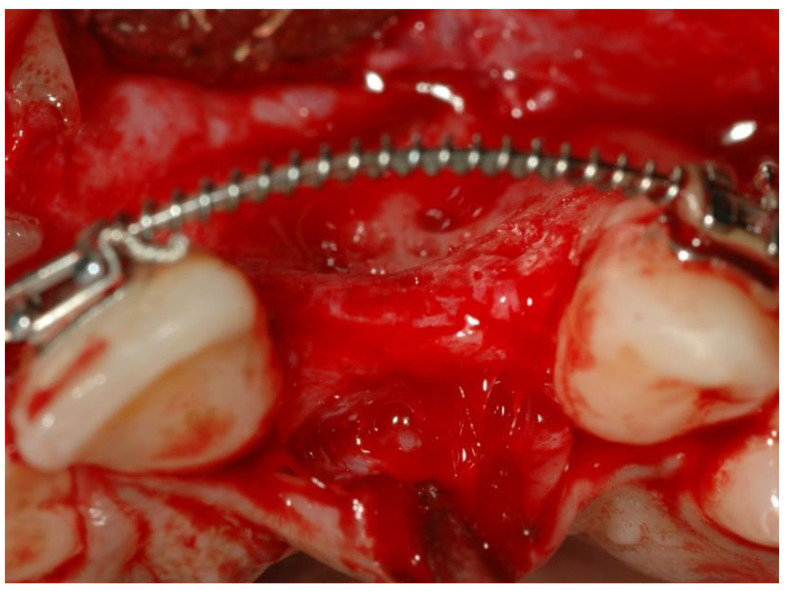
Deficient alveolar ridge.

The alveolar ridge was exposed to allow three-dimensional visualization of the defect (Figure 1).

**Figure 2:**
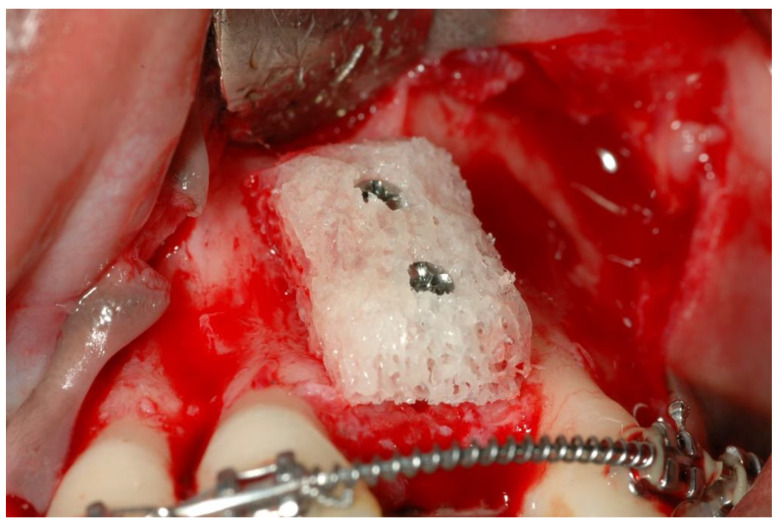
Cancellous bone-block fixation.

Cancellous block-grafts were refined to fit into the defect and fixed with bone screws. The sharp cortical edges were rounded and shaped to completely conform to the defect site (Figure 2).

**Figure 3:**
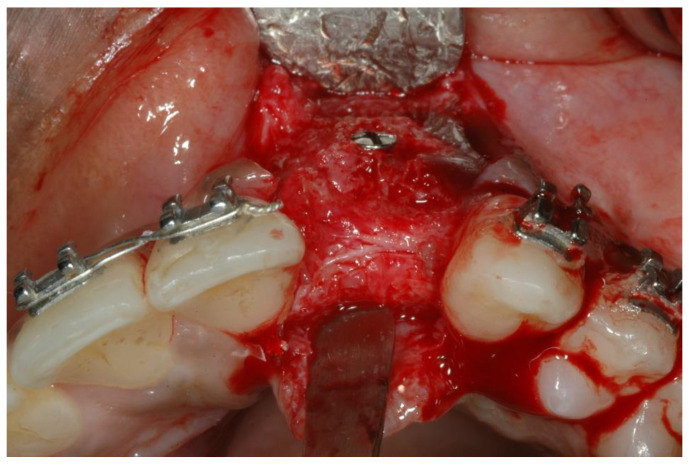
Block grafts incorporated into the surrounding bone.

A resorbable membrane was used to cover the block. The incision was closed using mattress sutures. Provisional restorations were modified to prevent the application of any pressure to the healing tissues. The grafted sites were allowed to heal. Access was obtained after 6 months via an incision similar to the one used during graft placement. Surgical exposure of the augmentation site revealed well-integrated block-grafts that were incorporated into the surrounding bone (Figure 3).

Rough surface titanium implants were placed (Mean length 12.33 mm; Mean diameter 4.02 mm).

**Prosthetic procedure**

A temporary abutment was adapted and a prefabricated acrylic was cemented. Occlusal scheme excluded lateral and occlusal contacts.

**Figure 4:**
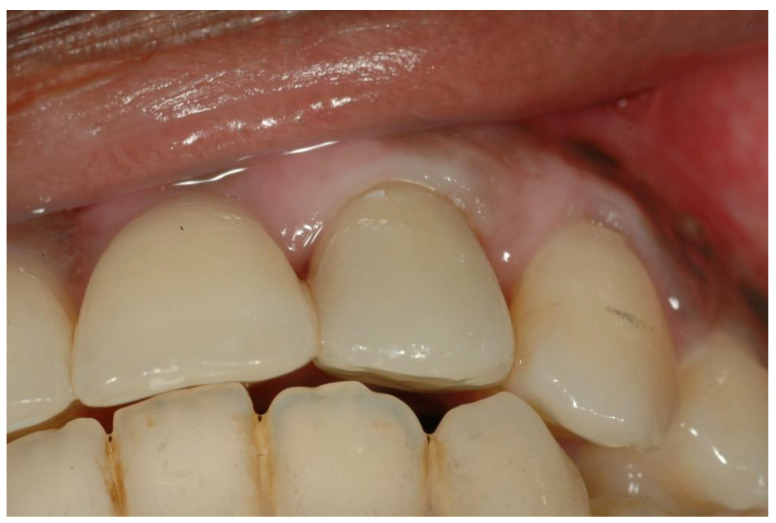
Restoration interocclusal relation.

Three months after implant placement, the temporary abutment and restoration were replaced with permanent ceramic abutment and cement retained fixed all ceramic prosthesis (Figure 4).

### 2.1. PES Index

Soft tissue evaluation (photographic visual assessment) was calculated using the PES index [3,4,5]. The mean of 2 evaluators (SN, LC) was used. PES is mainly affected by the local anatomy and surgical procedure used to repair the bone defects that routinely appear at the implant site after tooth extraction. This index examines 5 parameters: 1. mesial papilla, 2. distal papilla, 3. curvature of the facial mucosa, 4. buccal gingival height, 5. root/color curvature and soft tissue texture in the buccal aspect of the implant site. Each parameter can receive a score of 0, 1, 2 so the highest score that can be obtained in the index is 10 [3,4,5].

### 2.2. WES Index

In order to evaluate (photographic visual assessment) the restoration itself, the WES index was used, which is mainly influenced by the dental laboratory and prosthodontist instructions [3,4,5]. The mean of 2 evaluators (SN, LC) was used. As a reference, the natural homologous tooth was used. This index examines 5 parameters: 1. the original tooth shape, 2. the outline and volume of the clinical crown, 3. the overall color within it, the evaluation of hue and value indices, 4. the surface texture, 5. transparency and characterization. Each parameter receives a score of 0, 1, 2 and the highest result that can be obtained in the index is 10 [3,4,5]. The highest score that can be obtained in the PES / WES index is 20, which shows as high an esthetic fit as possible of the restored tooth on the implant and the soft tissues surrounding it. The value 6 is set to a total value for PES or WES that is defined as clinically acceptable, and thus the total value for PES / WES that is clinically acceptable is 12 [3].

### 2.3. Statistical Analysis

The statistical analysis was performed using SPSS software version 24.0 (SPSS Inc., Chicago, IL, USA; STATA 15.1, StataCorp LLC, College Station, TX, USA). A *p*-value < 0.05 was considered as statistically significant. Descriptive statistics were used for study participants and characteristics related to esthetic soft tissue and prosthetic rehabilitation analysis. Esthetic criteria (PES, WES) were averaged for statistical examination. An evaluation of patient demographics (age, gender, etiology, tooth involved, implant brand) as factors that may affect esthetic parameters was performed using multivariate analysis.

## 3. Results

The study included 25 patients (4 men and 21 women), with a mean age of 26.5 ± 9.92 years (age range 18–53 years). Implant location included tooth 21 (7/25 patients), tooth 22 (7/25 patients), tooth 12 (6/25 patients), tooth 11 (4/25 patients), tooth 23 (1/25 patients). The mean follow-up period was 65.56 ± 12.1 months (follow-up range 42–90 months) post- ISP delivery.

Severe bone loss was due to trauma (13/25); congenital (8/25); implant failure (2/25); endodontic treatment failure (1/25); and periodontal (1/25).

Implant brands were as follows—17/25 3I (Implant Innovations, West Palm Beach, FL), 7/25 MIS (MIS Implants Technologies, Bar Lev industrial center, Israel) and one implant Zimmer (Zimmer Biomet, Warsaw, IN, USA).

### 3.1. Esthetic Evaluation of Treatment Results Using PES/WES Indices

In the detailed table of PES and WES values (Table 1), the scores obtained in each of the parameters for 25 patients are presented. The mean value of PES + WES indices was 15.3 ± 2.85 (range 12–20). None of the 25 patients had a total PES + WES value <12 (the clinically acceptable defined threshold value) [3].

### 3.2. Analysis of Esthetic Results According to PES Values

**Figure 5:**
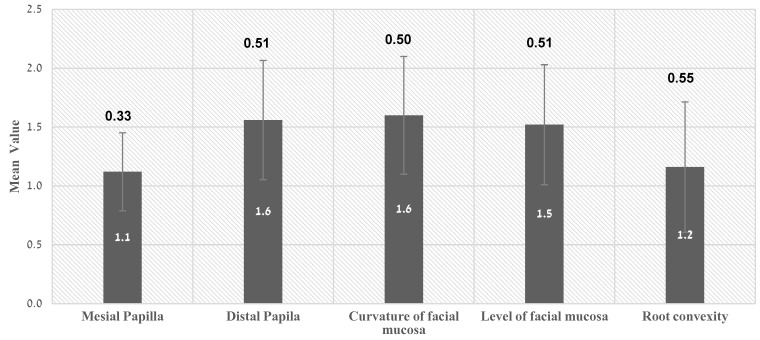
Mean PES value of each parameter.

The mean value of PES was 7 ± 1.74 (range 5–10) (Table 1). Figure 5 demonstrates that two parameters—the curvature of the facial mucosa (1.6 ± 0.50) and the level of facial mucosa (1.5 ± 0.51)—reached the high average scores. Root convexity (compatible with the buccal aspect of the implant site) presented the lowest mean values (1.2 ± 0.55). Only 6 of the 25 implant sites achieved the maximum value of 2 in this parameter (Table 1). For the papilla regions, mean values of 1.1 ± 0.33 for the mesial and 1.6 ± 0.51 for the distal papilla were obtained. The minimum threshold value defined as clinically acceptable for the PES or WES index is 6 [3]. In the present study, 17/25 ISPs (68%) had PES scores above the minimum threshold value, which generally indicates favorable esthetics (Table 1).

### 3.3. Analysis of Esthetic Results According to WES Values

**Figure 6:**
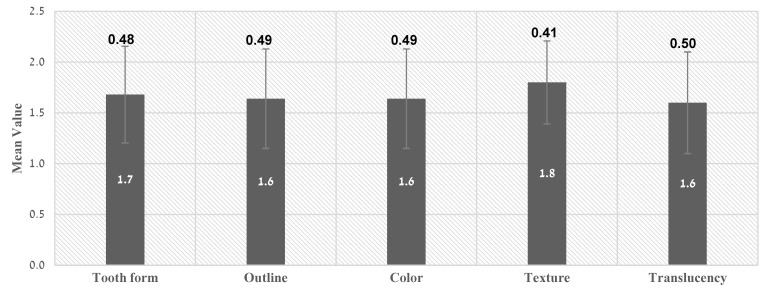
Mean WES value of each parameter.

The mean value of WES was 8.4 ± 2.12 (Table 1). Figure 6 demonstrates relatively similar results for all parameters examined: tooth form 1.7 ± 0.48, outline 1.6 ± 0.49, color 1.6 ± 0.49, texture 1.8 ± 0.41, and translucency 1.6 ± 0.50. Twenty-one (84%) crowns achieved a total WES value above 6 (the clinically acceptable value threshold) (Table 1).

**Figure 7:**
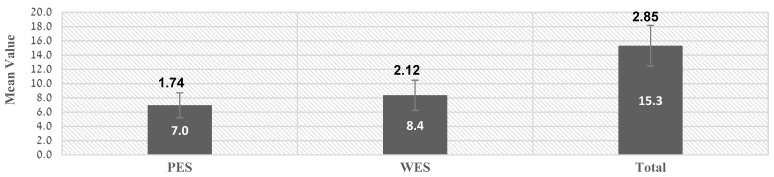
Mean total PES and WES value.

In the analysis of the combined esthetic results according to the PES and WES indices (Figure 7), total PES average (7 ± 1.74) was slightly lower than the WES average (8.4 ± 2.12) and the average of both was 15.3 ± 2.85.

### 3.4. Multivariate Analysis

Multivariate analysis evaluated patient demographics (age, gender, etiology, tooth involved, and implant brand) as factors that may affect esthetic parameters. There was no statistically significant effect (*p* > 0.05) on the esthetic parameters evaluated.

## 4. Discussion

This retrospective study presents an evaluation of the esthetic outcome of 25 patients who underwent bone augmentation using allogeneic cancellous bone-blocks, late implant placement, and immediate esthetic reconstruction in the anterior atrophic maxilla.

The anterior maxilla is a challenging area, with a high esthetics demand [22]. The bone support and soft tissue dimensions surrounding the restorative implants are key factors, related to each other, shaping the esthetic result [23,24]. The position of the dental implant in relation to neighboring teeth and the alveolar ridge are factors that affect the level of osseointegration over the years [24]. In order to maintain bone height and prevent gingival recession over time, verification of at least 2 mm with a preference for 4 mm of bone buccal to the implant neck is required [2,25,26]. Insufficient thickness of the buccal bone may lead to loss of buccal bone height, and soft tissue dehiscence that will lead to impairment of the biomechanical and esthetic result [27].

The prerequisite of 2–4 mm buccal bone motivated many clinicians to develop many augmentation techniques over the years. Some of them, such as the use of allograft bone-blocks, have been proven effective. Consequently, the requirement of such extensive bone could be overcome [28].

Immediate esthetic restoration may be defined as a situation in which at the end of implant placement a temporary restoration is installed. Immediate loading benefits include immediate function and esthetics, shortening treatment time, and avoiding a second surgical stage. In the case of a single tooth restoration, good adaptation of the gingiva is enabled and an esthetic result may be obtained more easily. Moreover, the accompanying function (within physiological boundaries) may encourage bone formation and contribute to better and faster osseointegration [17].

In 2005, the “pink esthetic score” was introduced by Fürhauser et al. [29] and focused on soft tissue aspects of anterior implants. In 2009, Belser et al. [3] presented the modified esthetic index PES/WES which is a renewed variation that incorporates the previous definitions along with the addition of the white esthetic index. The authors defined that the clinically acceptable total threshold for PES + WES is 12 [3,4].

**Figure 8:**
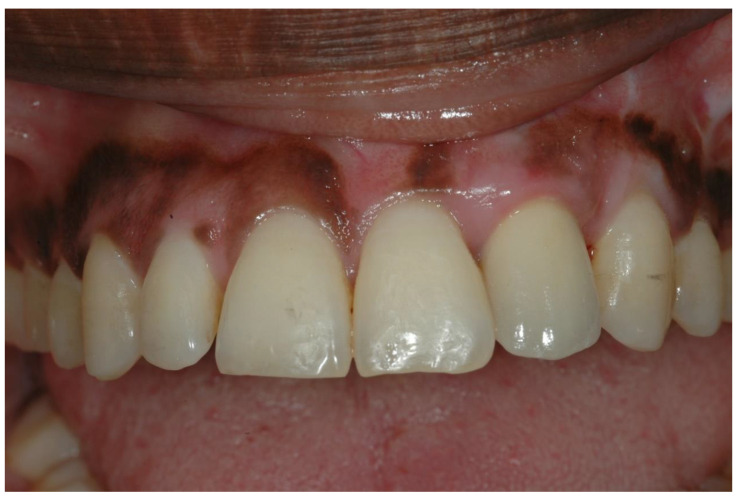
Tooth 22 was reconstructed. PES + WES score was16.

In the present study, the esthetic results were measured using PES and WES indices. The overall average score obtained in the PES + WES indices was 15.3 ± 2.85 (range 12–20), indicating a successful esthetic result. All patients included in the study (Figure 8) received a PES + WES value above 12, which supports the fact that the final esthetic result obtained in the study was successful and satisfactory (≥12) [3].

The minimum threshold value defined as clinically acceptable for a single index, PES or WES, is 6.3. The overall result obtained in this study in the PES index was 7 ± 1.74 (after a mean follow-up period of 65.56 ± 12.1 months) and is similar to the results obtained in the study of Yildis, P. et al. [30], who reported that in the “immediate loading” group on a one-year follow-up examination of the procedure, the result of the PES index averaged 8.2. In another study using the PES index of Raes et al. similar results were found (PES = 7.2) [31]. Another study [32] by Bjorn et al., (2020), in which immediate loading was performed in two study groups to test the esthetic level of the results, the results were similar (PES = 10.53/14, WES = 10.36/14) in a one-year follow-up period.

In a study [33] by Wessels, R. et al., (2020), cases were investigated in which, in addition to immediate implant placement and late loading, connective tissue grafting (CTG) was also performed. The overall PES result in the same study is 7.8 (at 5-year follow-up), which is quite similar to the result of the current study (PES = 7), although in the present study no soft tissue grafting was performed.

The results of the present study can be explained by the fact that thanks to the amount of buccal bone gained using allogeneic cancellous bone-block, the esthetic result was maintained for a long time (follow-up range 42–90 months). These results are consistent with the results of the study by Nissan et al., (2011) showing that after augmentation of an allograft bone-block in atrophic anterior maxilla an average bone gain of 5 ± 0.5 mm in the horizontal dimension and 2 ± 0.5 mm in the vertical dimension was achieved. Also, the buccal bone thickness observed near the neck of the implant was 2.5 ± 0.5 mm on average [16].

The mean value of the WES index in the present study was 8.4 ± 2.12 (Table 1). The WES index is mainly affected by the laboratory work and the prosthodontist instructions [3]. In the present study, one laboratory was used for all patients and the restoration was performed by one prosthodontist (permanent restoration of a metal-free crown) which ensured good standardization.

The results of the study demonstrate that a favorable esthetic result was obtained thanks to the use of a cancellous allogeneic block as a preliminary bone graft.

Limitations of the present study include: retrospective study, performed in only one treatment center, one augmentation material was used, and limited sample size. Further studies are recommended to test the effect of allogeneic bone-block grafting on the esthetic results following immediate esthetic restoration.

## 5. Conclusions

The use of a cancellous bone-block and immediate restoration allows achievement of a predictable and stable esthetic result of the soft and hard tissues surrounding ISP, which will be preserved over time (follow-up of 42–90 months).

## Figures and Tables

**Table 1:** Detailed PES and WES values of all patients.

			WES						PES					
Patient	Implant Site	Total PES + WES	Total WES	Trans-Lucency	Texture	Color	Outline	Tooth Form	Total PES	Root Convexity	Level of Facial Mucosa	Curvature of Facial Mucosa	Distal Papilla	Mesial Papilla
1	11	12	6	1	2	1	1	1	6	1	1	1	2	1
2	12	12	7	1	2	1	1	2	5	1	1	1	1	1
3	12	15	10	2	2	2	2	2	5	1	1	1	1	1
4	22	19	10	2	2	2	2	2	9	2	2	2	2	1
5	11	19	10	2	2	2	2	2	9	2	2	2	2	1
6	22	19	10	2	2	2	2	2	9	2	2	2	2	1
7	21	19	10	2	2	2	2	2	9	2	2	2	2	1
8	21	15	10	2	2	2	2	2	5	1	1	1	1	1
9	22	13	5	1	1	1	1	1	8	1	2	2	2	1
10	21	14	7	1	1	1	2	2	7	1	2	2	1	1
11	12	15	10	2	2	2	2	2	5	1	1	1	1	1
12	22	15	10	2	2	2	2	2	5	1	1	1	1	1
13	21	17	10	2	2	2	2	2	7	0	2	2	2	1
14	22	12	5	1	1	1	1	1	7	0	2	2	2	1
15	21	13	5	1	1	1	1	1	8	1	2	2	2	1
16	22	13	5	1	1	1	1	1	8	1	2	2	2	1
17	21	18	10	2	2	2	2	2	8	1	2	2	2	1
18	11	20	10	2	2	2	2	2	10	2	2	2	2	2
19	21	20	10	2	2	2	2	2	10	2	2	2	2	2
20	12	15	10	2	2	2	2	2	5	1	1	1	1	1
21	11	17	10	2	2	2	2	2	7	1	1	2	2	1
22	12	15	10	2	2	2	2	2	5	1	1	1	1	1
23	12	12	7	1	2	2	1	1	5	1	1	1	1	1
24	22	12	6	1	2	1	1	1	6	1	1	1	1	2
25	23	12	6	1	2	1	1	1	6	1	1	2	1	1
Average		15.3	8.4	1.6	1.8	1.6	1.6	1.7	7	1.2	1.5	1.6	1.6	1.1
SD		2.85	2.12	0.50	0.41	0.49	0.49	0.48	1.74	0.55	0.51	0.50	0.51	0.33

## Data Availability

Data supporting reported results can be found in the tables and figures.
